# Organizational Factors Moderating Changes in Tobacco Use Dependence Care Delivery Following a Comprehensive Tobacco-Free Workplace Intervention in Non-Profit Substance Use Treatment Centers

**DOI:** 10.3390/ijerph181910485

**Published:** 2021-10-06

**Authors:** Kathy Le, Tzuan A. Chen, Isabel Martinez Leal, Virmarie Correa-Fernández, Ezemenari M. Obasi, Bryce Kyburz, Teresa Williams, Kathleen Casey, Matthew Taing, Daniel P. O’Connor, Lorraine R. Reitzel

**Affiliations:** 1 Long School of Medicine, The University of Texas Health Science Center at San Antonio, San Antonio, TX 78229, USA; lek1@livemail.uthscsa.edu; 2 Department of Psychological, Health & Learning Sciences, The University of Houston, 3657 Cullen Blvd Stephen Power Farish Hall, Houston, TX 77204-5029, USA; tchen3@central.uh.edu (T.A.C.); imarti31@central.uh.edu (I.M.L.); vcorreaf@central.uh.edu (V.C.-F.); emobasi@uh.edu (E.M.O.); mtaing@central.uh.edu (M.T.); 3 HEALTH Research Institute, The University of Houston, 4849 Calhoun Rd., Houston, TX 77204, USA; 4 Integral Care, 1430 Collier Street, Austin, TX 78704, USA; bryce.kyburz@integralcare.org (B.K.); teresa.williams@integralcare.org (T.W.); kathleen.casey@integralcare.org (K.C.); 5 Department of Health & Human Performance, The University of Houston, 3875 Holman Street, Garrison Gymnasium, Room 104, Houston, TX 77204, USA; dpoconno@central.uh.edu

**Keywords:** smoking, implementation science, tobacco control, training, education, organizational moderators, readiness for change, tobacco use disorders, intervention, non-profit substance abuse treatment centers, substance use disorders

## Abstract

Although tobacco use is the leading preventable cause of death and is elevated among patients with substance use disorders, many substance use treatment centers (SUTCs) do not offer tobacco use interventions (i.e., screening and treatment). This study examined a key outcome of the implementation of a tobacco-free workplace program that provided education and specialized training to employees; namely, changes in clinician provision of the five As (Asking about tobacco use; Advising to quit; Assessing willingness to quit; Assisting with quitting; Arranging follow-up) from before to after the larger program implementation. The five As are a brief tobacco screening and treatment protocol that was taught as part of the program and that formed the basis for further intervention (e.g., provision of nicotine replacement therapies, Motivational Interviewing to enhance desire and willingness to make a quit attempt). Moreover, we also examined organizational moderators that may have impacted changes in the delivery of the five As over time among clinicians from 15 participating SUTCs. The number of the centers’ total and unique annual patient visits; full-time employees; and organizational readiness for implementing change were assessed as potential moderators of change in clinicians’ behaviors over time. Clinicians completed pre- and post-program implementation surveys assessing their provision of the five As. Results demonstrated significant increases in Asking (*p* = 0.0036), Advising (*p* = 0.0176), Assisting (*p* < 0.0001), and Arranging (*p* < 0.0001). SUTCs with higher Change Efficacy (*p* = 0.025) and lower Resource Availability (*p* = 0.019) had greater increases in Asking. SUTCs with lower Resource Availability had greater increases in Assessing (*p* = 0.010). These results help guide tobacco control program implementation to increase the provision of tobacco use interventions (i.e., the five As) to SUTC patients and elucidate Change Efficacy and Resource Availability as organizational factors promoting this clinician behavior change.

## 1. Introduction

Tobacco use has been associated with several types of cancers (e.g., lung, mouth, throat) and chronic cardiovascular and respiratory diseases [1]. It is also the leading preventable cause of death and disability in the United States [2]. While tobacco use rates have been gradually decreasing in the United States overall, these rates remain high among patients with substance use disorders. Specifically, whereas 14% of the general adult population smoke cigarettes, the rate of smoking among patients with substance use disorders is 65–87% [2,3]. Patients with substance use disorders are two to four times more likely to be a smoker than those without a substance use disorder; they are also more likely to die from their tobacco use than from the non-nicotine substance use disorder for which they sought treatment [4,5,6,7,8].

Many patients with substance use disorders are motivated to quit and are interested in being offered smoking cessation interventions, which include tobacco use screening and treatment, within the context of substance use treatment centers [9,10,11,12]. Unfortunately, many substance use treatment centers do not offer interventions for tobacco use disorder in the context of non-nicotine substance use intervention [13]. Other studies have cited that one reason for this is the prevailing clinical misconception among employees that quitting tobacco will derail the recovery of patients in non-nicotine substance use disorder treatment, specifically that it may be difficult or too stressful for patients to quit both successfully [14,15]. Counter to this misconception, tobacco use cessation may actually enhance substance use disorder treatment outcomes [16,17]. For instance, in one study, patients with substance use disorders who quit smoking in treatment maintained 5-year alcohol and other drug abstinence at higher rates than those patients who had not quit smoking, which is a finding consistent with other studies looking at short-term outcomes [18,19]. Likewise, quitting smoking may also improve psychiatric symptoms [20,21] and psychological quality of life [22], which is a critical need given that individuals with past or current substance use disorders frequently experience comorbid psychiatric and/or medical conditions [9]. Given the synergistic effects of tobacco and other drug use on health [9], the urgency of addressing tobacco-related disparities in substance use treatment settings is further compounded by the knowledge that over 50% of individuals with substance use disorders die from tobacco-related diseases, as opposed to those related to alcohol and other drug use [5]. Other reasons why tobacco use disorder has been largely ignored in substance use treatment centers include insufficient tobacco-related intervention training for clinicians, employee attitudes that hinder tobacco treatment (e.g., considering tobacco treatment a low priority), the cost of nicotine replacement therapy from both the patients’ and the centers’ perspectives, and high employee smoking rates [23,24,25,26,27]. This recognized need for tobacco intervention among patients with substance use disorders has precipitated clinical practice guidelines to routinely screen for and treat tobacco use among those with substance use disorders [28,29]. One mechanism to address the lack of accessible tobacco intervention available to patients with substance use disorders has been through the implementation of comprehensive, targeted tobacco control programs within substance use treatment centers [15,23].

*Taking Texas Tobacco Free* is an academic–community partnership that assists substance use treatment centers with the implementation of a comprehensive tobacco-free workplace program to build organizational capacity to address tobacco use dependence concurrently with other addictive substance use disorders. This tobacco-free workplace program entails the following: (1) tobacco-free workplace policy development and implementation, which includes the creation or re-release of a policy that forbids tobacco use on the grounds of, or in the buildings associated with, the treatment center that is accompanied by a plan for quality assurance/monitoring and handling incidents of non-compliance); (2) a 1–2 hour educational training session for employees (comprised of clinicians and non-patient-facing staff, wherein the attendees are taught about how to screen for and treat tobacco use, such as with the use of the five As as discussed below); (3) specialized training for select clinicians (Certified Tobacco Treatment Specialist training, basic Motivational Interviewing skills); (4) resource provision (e.g., nicotine replacement therapy, signage, passive dissemination materials); and (5) technical assistance during implementation. The dissemination and implementation of evidence-based tobacco control policies and practices have been shown to promote the clinician provision of tobacco intervention to patients and increase quit attempts among patients [13,29,30,31]. Thus, the focus of these efforts is to change clinician behavior to specifically increase their provision of evidenced-based interventions to patients in real-world practice settings, as it is understood that patients who receive more smoking cessation interventions from their clinicians are more likely to make a quit attempt and less likely to use tobacco during treatment [28,29,32].

*Taking Texas Tobacco Free* has been implemented throughout Texas in behavioral health treatment settings and substance use treatment centers [33,34,35,36,37,38,39]. Implementation outcome data have supported significant increases in tobacco-related knowledge among employees, increases in clinicians’ self-reported use of the five As, and increases in organizational capacity for tobacco intervention provision to patients [33,34,35,36,37,39,40,41]. The five As are an evidence-based, brief tobacco cessation intervention that has been recommended in clinical treatment guidelines [29,42]. They include Asking the patient about their tobacco use (tobacco screening) and the following tobacco treatment strategies: Advising them to quit, Assessing their willingness to quit, Assisting them with their quit attempt, and Arranging for follow-up. These would be implemented such that all patients should be Asked about (screened for) tobacco use, Advised to quit, and their willingness to make quit attempt should be Assessed. For those willing to make a quit attempt, they should be Assisted to do so through the provision of empirically-based interventions (e.g., medications/nicotine replacement therapies plus brief counseling), and follow-up to check on the patient’s progress should be Arranged. These steps should occur at every patient contact. While the five As were only one aspect of providing care for tobacco-using patients that clinicians were taught in *Taking Texas Tobacco Free*, they are considered a bedrock from which any further intervention actions (e.g., provision of nicotine replacement therapy, Motivational Interviewing to increase desire to quit) would arise. Therefore, their use by clinicians was considered a primary indicator of education/training effectiveness. Similar tobacco-free workplace programs that have been implemented within substance use treatment centers have been successful in improving attitudes toward treating tobacco dependence among substance use treatment center leadership and employees, increasing nicotine replacement therapy provision on-site at the treatment center, increasing clinician provision of tobacco dependence treatment to patients (e.g., providing recommended counseling and pharmacotherapy), and incorporating tobacco interventions into substance use disorder treatment [15,23,43,44].

Although tobacco control programs have been shown to be effective, it is currently unclear how and which organizational moderators impact the degree of adoption and program effectiveness among substance use treatment centers. In a prior study done with behavioral health treatment facilities, the number of unique patients served annually and the number of full-time employees moderated changes in clinicians’ delivery of the 5As to patients [37]. Apart from center demographics, center readiness also moderated changes in clinician behavior in that study. Specifically, belief that employees could take the steps for change (i.e., Change Efficacy), that employees had motivation to implement the change (i.e., Change Commitment), that employees had knowledge about the requirements for change (i.e., Task Knowledge), and that employees perceived that resources needed to implement a change were available (i.e., Resource Availability) moderated these changes [37].

However, while some organizational moderators have been delineated for tobacco control program implementation among non-profit behavioral health treatment facilities, the particular organizational moderators impacting tobacco control program implementation among non-profit substance use treatment centers may differ and thus warrant a separate exploration. Although both non-profit behavioral health treatment facilities and non-profit substance use treatment centers share similarities, such as their diverse patient population and their provision of care to patients regardless of patients’ ability to pay, there are significant differences between the two organization types. Behavioral health treatment facilities see a greater number of unique patients annually and employ a greater number of employees [33,37,45,46,47]. Additionally, substance use treatment centers may treat more specialized patient populations than behavioral health treatment facilities: while behavioral health facilities may broadly serve adults or children [48], substance use treatment centers may serve specific patient populations (e.g., patients who are sexual minorities, women and their children, patients with a disability) or patients with specific use disorders (e.g., opioid use) [38,49,50,51]. Results elucidating the organizational moderators impacting tobacco control program implementation among substance use treatment centers, specifically, can be used to optimize the implementation and maintenance of tobacco-free workplace programs within these settings. This is especially important to understand as there is a prominent research-to-practice translation gap in substance use treatment centers whereby evidence-based interventions for tobacco cessation are not being consistently implemented [52]. Moreover, this research can help address tobacco use and tobacco-related disease inequities among subpopulations with high tobacco use rates as well as help respond to calls to implement evidence-based tobacco control programs and community interventions in order to reduce smoking and remove it as a public health issue in the US [53].

## 2. Materials and Methods

### 2.1. Study Aims

The purpose of the current study was to examine changes from pre- to post- implementation in clinician provision of the five As. Additionally, the organizational-level factors, specifically organizational demographics and organizational readiness for change, that moderate clinician provision of the five As for tobacco use disorder from pre- to post-*Taking Texas Tobacco Free* program implementation were examined.

### 2.2. Characteristics of Participating Substance Use Treatment Centers

In total, 19 non-profit substance use treatment centers were enrolled in the *Taking Texas Tobacco Free* program from December 2017 to May 2020. Recruitment of substance use treatment centers was primarily accomplished through direct email solicitation to each respective center’s CEO and/or word of mouth from other substance use treatment centers. While program enrollment occurred on a rolling basis over 3 years, each participating center was provided all components of the *Taking Texas Tobacco Free* program and followed similar implementation timelines. The rolling recruitment of centers over the 3 years facilitated the ability of the *Taking Texas Tobacco Free* program to intervene across Texas (the largest state in the continental US) and allowed centers to start work with us at times that would be most convenient for them with respect to other center initiatives and priorities.

Of the 19 substance use treatment centers enrolled, 4 withdrew from the study prior to completion of the post-implementation measures. Consequently, this study discusses results from the remaining 15 participating substance use treatment centers. Of the 4 substance use treatment centers that withdrew, 1 center did not provide center demographics information. Of the remaining 3 centers, center demographics information does not appear to meaningfully vary from the remaining 15 centers that did participate apart from 1 center having no full-time employees. Although there does not seem to be a clear pattern between substance use treatment centers that withdrew, reported reasons for withdrawing among the 4 centers included competing demands, competing priorities due to the pandemic, and concern that the implementation of a completely tobacco-free workplace policy would negatively impact their patient census. The team provided these centers with testimonials and empirical data to address fear in reduced census, but it was to no effect.

Altogether, the 15 completing substance use treatment centers reported 850 employees (including clinicians and non-patient-facing (general) employees) at implementation. These substance use treatment centers served a total of 82,927 unique patients through 299,267 annual visits, as per their recent annual reports. Of these substance use treatment centers, many served unique populations, such as individuals who are experiencing homelessness, vulnerably housed, pregnant, and/or involved with the criminal justice system. These characteristics of the substance use treatment centers are described further in a separate study [41].

### 2.3. Program Implementation

Approval of all study procedures was obtained by the IRB at the University of Houston (STUDY00000472, approval date 27 July 2017). A Memorandum of Understanding, which outlined the overall program requirements and responsibilities, was signed by the CEO (or their designee) of participating substance use treatment centers. Signing this memorandum marked official enrollment in the program. Thereafter, the full *Taking Texas Tobacco Free* program was implemented over a 7.2–13.6-month period (mean = 10.96, SD = 3.84). Implementation timeframes differed between centers based on their respective capacity to fully participate in each component of the program, which might differ based on concurring initiatives, intervening events (e.g., recovery from natural events such as hurricanes), reduced operating hours (e.g., during the pandemic), ability to send groups of employees to educational programming while still providing care to patients, etc. Thus, the timeline was tailored in partnership with the center’s leadership.

Implementation included a 1–2 hour tobacco education training discussing the health risks of tobacco use; benefits of tobacco use treatment for patients with substance use disorders; the use of the five As for screening and treating tobacco use; and specific tobacco treatment options (e.g., nicotine replacement therapy, pharmacotherapy, counseling). Regardless of the clientele or specificity of the non-nicotine substance use treatment provided within the participating centers, the training of clinicians on the use of the five As was invariant, as too was its intended application across patients. The *Taking Texas Tobacco Free* program also sponsored clinicians to obtain specialized trainings (i.e., Certified Tobacco Treatment Specialist training and Motivational Interviewing); these components of the program are detailed elsewhere [33,34,35,38,39,40]. Each participating center could avail themselves of these specialized trainings, which had a much broader scope than training on the five As. Importantly, while specialized trainings were offered, they were not focused on the five As, and thus, all participating centers received uniform training on the five As. Involved centers did not receive monetary compensation for their participation in the program, although they received various resources without cost (e.g., passive dissemination materials, a starter kit of nicotine replacement therapy).

### 2.4. Procedures and Measures

#### 2.4.1. Organizational Demographics and Readiness for Implementing Change

Center leadership (e.g., CEOs or their designees) provided information on their substance use treatment center’s patient demographics, organizational characteristics, and readiness for change within ≈2 weeks following enrollment via an online survey. Organizational characteristics measured included the center’s number of total annual patient visits, unique annual patient visits, and full-time employees. The Organizational Readiness for Implementing Change (ORIC) scale [54] was used to assess readiness for change. The ORIC includes 24 items that comprise a total score (α = 0.73) and 5 subscale scores: (1) Resource Availability, “We have the expertise to implement this change,” α = 0.61; (2) Change Efficacy, “People who work here feel confident that the organization can support people as they adjust to this change,” α = 0.93; (3) Change Valance, “We believe this change will benefit our community,” α = 0.00 (alpha reflects almost no variation in responses); (4) Change Commitment, “People who work here are determined to implement this change,” α = 0.86; and (5) Task Knowledge, “We know what resources we need to implement this change,” α = 0.66. The total ORIC mean scores and the ORIC subscale mean scores ranged from 1 to 5. For analysis, these mean scores were used. Greater ORIC scores are associated with greater readiness for organizational change [55].

#### 2.4.2. Clinician Tobacco Screening and Treatment Behaviors

An investigator-generated survey was administered online to clinicians within each participating substance use treatment center following receipt of the information in Section 2.4.1 but before program implementation. Likewise, a similar survey was administered after each aspect of the program (e.g., policy implementation, training) was administered within a respective center. This survey anonymously assessed clinicians’ use of the five As in screening (i.e., Asking about tobacco use) and in addressing tobacco use (i.e., Advising users to quit, Assessing interest in quitting, Assisting patients with a quit attempt, and Arranging follow-up to assess progress). Surveys included a cover letter that provided information on the purpose of the study, purpose of the survey, and the voluntary nature of their participation. An anonymous approach to data collection was used to maintain the privacy of respondents and to encourage honest responses about their provision of care to patients. Since this was a workplace intervention, it is possible that identifiable data collection would have introduced risk, or the perceptions of risk, of occupational repercussions for a failure to provide evidence-based tobacco use care to patients that might have led to non-response or inaccurate responses. However, based on this methodology, pre- and post-implementation data could not be matched at the clinician level. Items included the following: Ask (“In your clinical work here last month, did you ask patients about their smoking status?”); Advise (“With regard to patients that you saw last month who smoked, did you advise them to quit smoking?”; Assess (“With regard to patients that you saw last month who smoked, did you assess their willingness to make a quit attempt?”); Assist (“With regard to patients that you saw last month who smoked, did you assist them to quit by providing treatment or making a referral for treatment?”); and Arrange (“With regard to patients that you saw last month who smoked, did you arrange to follow up with them to assess their progress regarding smoking cessation?”). Response options for these questions were coded as No = 0 and Yes = 1. Potential respondents were given ≈3 weeks to complete these measures, and reminders for their completion were sent each week during that timeframe.

### 2.5. Statistical Analysis

Changes in the delivery of the five As from pre- to post-implementation were assessed separately for each substance use treatment center and for all substance use treatment centers combined. First, Chi-square tests or Fisher exact tests were conducted, as appropriate, to examine pre- and post-implementation changes in the five As with unmatched data at the clinician level. Next, the potentially moderating effects of (1) median-split organizational demographics (e.g., number of total annual patient visits, unique annual patient visits, and full-time employees) and (2) readiness to change (via ORIC subscales) on changes in the delivery of the five As were examined with an interaction term in their respective analysis. ORIC subscales were mean-centered prior to moderation analyses. Tests of moderation were examined in covariate-adjusted models. In adjusted moderation models of each organizational demographics variable, covariates included the overall ORIC score and other organizational demographics. In adjusted moderation models of the ORIC subscales, covariates included each of the three organizational demographic variables. The nested data structure of clinicians within the substance use treatment centers was accounted for through generalized linear mixed models (generalized linear mixed model, binomial distribution, logit link, and variance components for the variance matrix). All statistical analyses were conducted using SAS version 9.4 [56] with alpha set at 0.05.

## 3. Results

### 3.1. Organizational Demographics

Of the 15 participating substance use treatment centers, the median (range) number of total annual patient visits, unique annual patient visits, and full-time employees was 7825 (range: 535–101,869), 385 (range: 45–64,419), and 19 (range: 5–304), respectively. ORIC means (± SD) were as follows: Change Efficacy (4.85 ± 0.31, in-sample range: 4–5), Change Commitment (4.71 ± 0.37, range: 4–5), Task Knowledge (4.24 ± 0.58, range: 4–5), Resource Availability (4.25 ± 0.53, range: 4–5), Change Valence (4.99 ± 0.05, range: 4–5), and overall ORIC (4.67 ± 0.19, range: 4–5). The ORIC subscale mean scores ranged from 1 to 5 where 5 is associated with greater readiness for organizational change.

### 3.2. Pre- to Post-Implementation Change in Clinician Screening and Treatment Behaviors

From pre- to post-program implementation, clinicians reported significant increases in each component of the five As except Assess: Ask: 66.25% to 77.84%, *p* = 0.0036; Advise: 60.54% to 71.82%, *p* = 0.0176; Assess: 70.98% to 78.80%, *p* = 0.0712; Assist: 45.95% to 69.61%, *p* < 0.0001; and Arrange: 35.14% to 59.89%, *p* < 0.0001). Notably, Assist and Arrange had the lowest rates of provision at both pre- and post-implementation. See Supplementary Table S1 for detailed information.

### 3.3. Organizational Demographics as Moderators of Changes in Clinician Screening and Treatment Behaviors

None of the organizational demographics (i.e., number of total annual patient visits, unique annual patient visits, and full-time employees) were significant moderators for change in the delivery of any of the five As, from pre- to post-implementation, in analyses adjusted for readiness to change (see Supplementary Table S2).

### 3.4. Organizational Readiness for Change as Moderators of Changes in Clinician Screening and Treatment Behaviors

In adjusted analyses, the moderating effects of Change Efficacy (γ = 1.638, *SE* = 0.728, *p* = 0.025) and Resource Availability (γ = −1.666, *SE* = 0.710, *p* = 0.019) were significant in changes in Asking patients about smoking status over time. Likewise, Resource Availability was also a significant moderator in Assessing willingness to quit (γ = −1.923, *SE* = 0.747, *p* = 0.010). Examination of these significant moderating effects suggest that substance use treatment centers with higher initial efficacy for change were associated with greater increase in Asking behavior. However, substance use treatment centers with lower baseline resources had greater increases in Asking about smoking and Assessing willingness to quit relative to those substance use treatment centers reporting greater initial resources (see interaction terms in Table 1; full model results are available in Supplementary Table S3).

Further analyses specifically showed that at pre-implementation, 74.03% of clinicians from centers with high Resource Availability Asked about tobacco use (compared to 52.38% of those from low Resource Availability centers) and 81.25% Assessed willingness to quit (compared to 57.29% of those from low Resource Availability centers). By post-implementation, rates of Asking (76.11% in high Resource Availability centers and 80.25% in low Resource Availability centers) and Assessing (77.14% in high Resource Availability centers and 81.01% in low Resource Availability centers) were more equitable between groups. Chi-square tests supported significant differences between pre- and post-implementation Asking and Assessing for clinicians from substance use treatment centers with low Resource Availability (Asking: X^2^ = 15.51, *p* < 0.0001; Assessing: X^2^ = 11.21, *p* = 0.0008). However, no significant differences were found among clinicians from substance use treatment centers with high Resource Availability (Asking: X^2^ = 0.15, *p* = 0.70; Assessing: X^2^ = 0.59, *p* = 0.44).

## 4. Discussion

Results from the current study indicated that clinicians from 15 substance use treatment centers in Texas, US, increased their provision of each of the five As of tobacco intervention (i.e., screening and treatment) from before to after the implementation of a comprehensive tobacco-free workplace program. For each of the five As, except for Assessing interest in quitting smoking, changes in tobacco intervention provision were statistically significant. In the case of Assess, pre- to post-implementation results may not have been significant due to high levels of clinicians Assessing willingness to quit tobacco at baseline; of the five As, Assess had the highest level of baseline provision (70.98%). While it is unclear why Assess had the highest rate of provision at baseline, it is possible that after Asking patients about their tobacco use, clinicians may have chosen to skip this aspect of the five As on repeat visits from the same patient and began instead with Assessing their willingness to quit on each subsequent visit. Overall, these findings support the feasibility of changing clinicians’ tobacco intervention behaviors in regard to their substance use disorder patients following a comprehensive, evidence-based tobacco control program. This tobacco control program included policy changes, training, resource provision, and technical support. Other studies suggest that increasing clinicians’ provision of evidence-based interventions may increase quit attempts and promote reduced tobacco use during treatment among patients with substance use disorders who smoke [28,32]. Thus, the present results add to the literature supporting the implementation of these programs to expand a substance use treatment center’s capacity to screen for and intervene on tobacco use, which may ultimately affect the tobacco use and tobacco-related disease inequities among individuals with substance use disorders [15,23,33,34,35,36,37,39,40,41,44]. Results also demonstrated that organizational readiness for change moderated these changes in intervention delivery; specifically, substance use treatment centers with greater initial Change Efficacy demonstrated greater increases in Asking about tobacco use, whereas substance use treatment centers with lower Resource Availability demonstrated greater increases in Asking about tobacco use as well as Assessing willingness to quit tobacco. These results suggest that prior to the implementation of tobacco control programs, center leadership should be proactive in ensuring their employees are confident that the organization will support their ability to provide tobacco interventions and clearly communicate the availability of resources to accommodate the change.

The purpose of the *Taking Texas Tobacco Free* program was to expand capacity for treating tobacco use in substance use treatment settings through the introduction of several empirically supported tobacco control measures, including clinician education. Prior studies have shown that clinician education in tobacco interventions translates to increased provision of these services [57,58], and our results add to this literature. In this study, clinicians’ consistent provision of each of the tobacco intervention behaviors with their patients increased over time. Based on the number of patients seen at these substance use treatment centers annually, intervening to Ask about tobacco use alone—if done with every patient as intended—would impact the 82,927 patients seen therein. Of those 82,927 patients, about 65–87% (based on estimates of comorbid tobacco and other substance use disorders) are likely tobacco users [3]. However, results also highlight areas in need of improvement. Of the five As, Assist and Arrange have consistently been shown in the literature as having low rates of provision [37,42] as was the case in the current study. This may be because Ask and Advise are behaviors that are more easily supported within existing clinical infrastructure and are relatively simpler to execute; conversely, Assist and Arrange are more complex and require more coordination [58]. Additionally, Arranging follow-up was the lowest frequency behavior among participating clinicians in this study, as has been the case in prior studies [37,58], which is potentially due to inadequate reimbursement for this practice [58]. However, the reason for this pattern in the current work was unable to be ascertained from the data collected. Future studies should further explore the barriers and enablers of Assisting and Arranging that might impact the provision of these aspects of tobacco intervention. Some of these barriers and enablers might include incentives (e.g., reimbursement for all aspects of the five As), specialized training for Assisting and Arranging, and/or increased clinical preparations to accommodate Assisting and Arranging.

An important contribution of the current study was the investigation of how organizational readiness for change was associated with changes in clinicians’ intervention behaviors over time following implementation of a tobacco-free workplace program. This is important because these results can help guide tobacco control implementation efforts in similar settings and address the research-to-practice translation gap among substance use treatment centers [52]. In the current study, substance use treatment centers with greater Change Efficacy demonstrated significantly greater improvements in Asking their patients about tobacco use. This may be interpreted to suggest that leadership’s confidence in their clinicians’ ability to execute change was well-placed, as the results demonstrated their clinicians were quite capable of increasing provision of tobacco screening with training. It may be that leadership had seen their employees pivot to execute other screening initiatives in the past and expected that current efforts would yield a similar result. Alternatively, although speculative at this point, it may be that having leaders who were more confident they could support the change and the tasks required to affect the change did *something* (e.g., communicated that confidence or expectation) that resulted in clinicians being more resolved to deliver tobacco screening through Asking. As screening is the first step of tobacco intervention provision and clinicians may not pursue the first step of tobacco intervention if they do not feel confident in being able to provide subsequent steps of treatment [52,53], expression of leadership’s confidence that clinicians can change their practices in this area may help reinforce clinicians’ own self-efficacy and encourage both screening and subsequent treatment practice changes. Consequently, as center leadership prepare to implement tobacco control programs, they may wish to clearly link the initiative to prior success experiences when rolling it out to clinicians in order to build clinicians’ confidence in making the change and in having the support of the organization in doing so [42,57,58].

Another moderator of clinician intervention delivery in this study was Resource Availability, or an organization’s perception of the availability of expertise, skills, time, and other resources required to implement a change. Interestingly, substance use treatment centers with higher Resource Availability demonstrated significantly less improvements in Asking about tobacco use and Assessing interest in quitting behavior over time relative to centers with lower Resource Availability. Other research has suggested that greater Resource Availability, particularly of treatment resources, has positively impacted the implementation of evidence-based tobacco control programs [30,45]. Additionally, common issues brought up by clinicians that prohibit the delivery of tobacco cessation interventions have been lack of resources, including low access to financial support to provide tobacco treatment [43,59]. Thus, our results appear to contradict what prior research might suggest. However, a closer look at our data indicated that substance use treatment centers with higher Resource Availability were already Asking and Assessing at higher rates than those with lower Resource Availability at pre-implementation (see Results, Section 3.4). Thus, our results suggest that organizations with lower Resource Availability implementing a comprehensive tobacco-free workplace program may have more room for growth in the provision of tobacco-related interventions relative to their counterparts with more resources. This finding is important, as it suggests that low resource settings may have the most to gain from capacity-building programs such as *Taking Texas Tobacco Free*. Consequently, the perception of limited resources should not be a deterrent for the implementation of comprehensive tobacco-free programs.

Among centers with high Resource Availability, the changes in Asking and Assessing were not significant. This suggests that while well-resourced centers may still have the potential to broaden their capacity to provide tobacco interventions, additional research is needed to delineate how program implementation should be tailored for these centers to maximize behavior change. Interestingly, prior research on clinician behavior change in behavioral health treatment facilities implementing *Taking Texas Tobacco Free* have indicated similar patterns of high organizational readiness subscales demonstrating less growth in delivery of the five As over time. That study proposed this discrepancy could be due to an overconfidence in organizational readiness reported from center leadership that can negatively impact program adoption [37]. This may also explain the current pattern of results. That is, these results may represent a disconnection between the center leadership’s assessment of an organization’s readiness and the true readiness of the organization or of the employees’ assessments [37]. Stated differently, higher reported Resource Availability may reflect an overestimation of the tools and resources available for clinicians than is truly available or communicated. Consequently, future research should investigate mechanisms through which the perceptions of the leadership and employees regarding Resource Availability at their center can be better aligned. Nevertheless, it is also important to recognize that well-resourced centers may still benefit from the implementation of a tobacco-free workplace and tobacco education training. Although potential benefits of participation for these high Resource Availability centers were not statistically significant, they may have clinical significance.

In this study, no organizational demographic variable assessed (i.e., total annual patient visits, unique annual patient visits, and number of full-time employees) was a moderator of changes in clinicians’ delivery of the five As to their substance use disorder patients over time. This pattern of results differs from prior research conducted in behavioral treatment health facilities in Texas, US, where facilities with a lower number of unique patient visits and a lower number of full-time employees saw greater improvements in intervention delivery [37]. Proposed reasons for those results included the following: smaller organizations were better able to adopt the *Taking Texas Tobacco Free* program due to greater leadership involvement and support; reduced resistance from employees; and increased time for tobacco treatment in the context of potentially lower caseloads. Other studies have suggested that larger organizations are better able to implement change given their potential for having greater infrastructure and resources [60,61]; thus, delivery of the five As, in the context of this literature, might be expected to be greater in larger organizations. However, in the current study, the lack of relationship between organizational demographics and change in delivery of the five As implies that changing tobacco intervention behaviors among clinicians within substance use treatment centers might have nuances that may not lend itself to being moderated by organizational size. Additionally, our approach to assessing these potential moderators was specific to our sample, given that a median split procedure was used. This approach was selected for consistency with prior work [37]; however, differences between the participating centers’ demographics in these studies were sizeable, as behavioral health centers serve many more patients and have many more employees than substance use treatment centers. Thus, the relative restriction in range within the substance use treatment center’s demographics versus those found within behavioral health centers may have contributed to contrasting results. Future work in this area might consider using continuous organizational demographic moderators.

This study has limitations including its conduct only in Texas, which may preclude the generalization of results to substance use treatment centers elsewhere. Additionally, the results do not represent centers that were more likely to drop out or less likely to enroll in the tobacco-free workplace program that underlies this work. Clinician provision of the five As were self-reported; consequently, it is possible that clinicians over-reported or under-reported use of the five As. Moreover, the collection of data regarding clinicians’ behaviors “over the previous 30 days” (versus daily) may have increased the possibility of recall bias. Clinician survey responses were also collected anonymously, which precluded matched pre- and post-implementation comparisons of tobacco intervention delivery at the clinician level. As the ORIC was completed by center leadership, the ORIC results for each center reflect primarily the perspective of the center’s CEO or their designee. While ORIC items asked center leadership to assume the perspectives of their employees (“We have…” or “People who work here…”), future studies would benefit from integrating the perspectives of center employees. Moreover, the ORIC scores in this study were generally high (e.g., each was >4 on a scale that maxed at 5). This is expected given that the participating centers were early adopters of the program, but the high scores could also have also been influenced by the CEOs’ potential over-estimation of a climate of readiness for change amongst employees. Future studies with middle or late adopters, or that include the perspectives of various center stakeholders, may produce different results. Finally, while the study includes a discussion on organizational moderators that impact changes in clinician behaviors, it does not delve into the exact mechanisms through which they may do so. Additionally, this study does not include patient perspectives, verification of screening/treatment received, or data on quit attempts or abstinence. Although clinicians’ delivery of the five As was supposed to be invariant across patients and time, data were not collected about how this screening and intervention might have been affected by the patients’ non-nicotine substance use disorder, phase of recovery, etc., which might be of interest to report in future work.

## 5. Conclusions

Results from this study demonstrated that changes in clinicians’ delivery of the five As from pre- to post-implementation were significant in each domain except Assess. There is a need for additional research on barriers and enablers of Assisting and Arranging behaviors, which may include financial incentives to clinicians as an enabler, the need for specialized training for Assisting and Arranging, and/or increased clinical preparations to accommodate these clinician behaviors. Organizational readiness subscales (i.e., Change Efficacy and Resource Availability) were found to be significant moderators of changes in clinicians’ delivery of the five As to patients; organizational demographics were not. Center leadership should seek to increase clinician confidence in their ability to screen and treat tobacco regularly in clinical encounters with patients. This may entail additional trainings and/or leaderships’ explicit endorsement of confidence in their clinicians’ ability to address the change. Centers with lower initial Resource Availability should not be deterred from implementing a tobacco-free workplace program, as more gains in evidence-based care for tobacco use may be realized for them relative to their better resourced counterparts. However, there may be a discrepancy between the leaders’ and employees’ perceptions of available resources at their center. Consequently, center leaders should consider mechanisms by which they can assess and address their employees’ perceived preparation, perception of the organization’s readiness, and understanding of available of tools and resources that can be used to pursue the delivery of tobacco use disorder interventions.

## Figures and Tables

**Table 1:** Adjusted model of organizational readiness for change subscales as moderators of clinician screening and treatment behaviors pre- to post-program implementation.

Clinician Behaviors		ORIC Change Efficacy		
	Effect	Estimate	SE	*p*
Ask	Time (ref: pre-implementation)	0.815	0.239	0.001
	ORIC subscale	0.023	0.949	0.981
	ORIC subscale*time	1.638	0.728	0.025
Advise	Time (ref: pre-implementation)	0.639	0.230	0.006
	ORIC subscale	1.924	0.894	0.032
	ORIC subscale*time	0.272	0.702	0.698
Assess	Time (ref: pre-implementation)	0.569	0.252	0.024
	ORIC subscale	0.695	1.135	0.541
	ORIC subscale*time	0.440	0.784	0.574
Assist	Time (ref: pre-implementation)	1.190	0.227	<0.001
	ORIC subscale	0.102	0.711	0.886
	ORIC subscale*time	0.705	0.722	0.329
Arrange	Time (ref: pre-implementation)	1.260	0.229	<0.001
	ORIC subscale	2.287	0.843	0.007
	ORIC subscale*time	−1.292	0.808	0.110
**Clinician** **Behaviors**		**ORIC Change Commitment**		
	**Effect**	**Estimate**	**SE**	***p***
Ask	Time (ref: pre-implementation)	0.719	0.245	0.004
	ORIC subscale	2.918	0.650	<0.001
	ORIC subscale*time	−0.115	0.707	0.871
Advise	Time (ref: pre-implementation)	0.677	0.236	0.004
	ORIC subscale	2.124	0.603	<0.001
	ORIC subscale*time	0.564	0.656	0.391
Assess	Time (ref: pre-implementation)	0.491	0.261	0.060
	ORIC subscale	1.720	1.203	0.154
	ORIC subscale*time	-0.581	0.717	0.418
Assist	Time (ref: pre-implementation)	1.158	0.226	<0.001
	ORIC subscale	1.257	0.581	0.031
	ORIC subscale*time	−0.347	0.617	0.574
Arrange	Time (ref: pre-implementation)	1.177	0.220	<0.001
	ORIC subscale	1.761	0.586	0.003
	ORIC subscale*time	−0.698	0.594	0.241
**Clinician** **Behaviors**		**ORIC Task Knowledge**		
	**Effect**	**Estimate**	**SE**	***p***
Ask	Time (ref: pre-implementation)	0.802	0.244	0.001
	ORIC subscale	−1.021	0.349	0.004
	ORIC subscale*time	−0.658	0.605	0.278
Advise	Time (ref: pre-implementation)	0.689	0.234	0.004
	ORIC subscale	−0.087	0.520	0.868
	ORIC subscale*time	−1.096	0.563	0.052
Assess	Time (ref: pre-implementation)	0.509	0.258	0.049
	ORIC subscale	−1.515	0.476	0.002
	ORIC subscale*time	0.350	0.616	0.570
Assist	Time (ref: pre-implementation)	1.178	0.225	<0.001
	ORIC subscale	−0.231	0.325	0.477
	ORIC subscale*time	−0.474	0.509	0.353
Arrange	Time (ref: pre-implementation)	1.187	0.223	<0.001
	ORIC subscale	−0.319	0.422	0.450
	ORIC subscale*time	-0.112	0.499	0.822
**Clinician** **Behaviors**		**ORIC Resource Availability**		
	**Effect**	**Estimate**	**SE**	***p***
Ask	Time (ref: pre-implementation)	0.791	0.239	0.001
	ORIC subscale	0.645	0.538	0.232
	ORIC subscale*time	−1.666	0.710	0.019
Advise	Time (ref: pre-implementation)	0.656	0.231	0.005
	ORIC subscale	0.400	0.518	0.441
	ORIC subscale*time	−1.033	0.648	0.112
Assess	Time (ref: pre-implementation)	0.585	0.259	0.024
	ORIC subscale	0.758	0.615	0.218
	ORIC subscale*time	−1.923	0.747	0.010
Assist	Time (ref: pre-implementation)	1.188	0.226	<0.001
	ORIC subscale	0.899	0.391	0.022
	ORIC subscale*time	−0.963	0.573	0.093
Arrange	Time (ref: pre-implementation)	1.208	0.223	<0.001
	ORIC subscale	0.652	0.413	0.115
	ORIC subscale*time	−0.366	0.544	0.502
**Clinician** **Behaviors**		**ORIC Change Valence**		
	**Effect**	**Estimate**	**SE**	***p***
Ask	Time (ref: pre-implementation)	0.698	0.236	0.003
	ORIC subscale	6.488	3.282	0.049
	ORIC subscale*time	−2.930	2.452	0.233
Advise	Time (ref: pre-implementation)	0.648	0.232	0.005
	ORIC subscale	3.686	3.783	0.331
	ORIC subscale*time	1.029	2.416	0.670
Assess	Time (ref: pre-implementation)	0.474	0.256	0.065
	ORIC subscale	6.724	4.181	0.109
	ORIC subscale*time	−3.745	2.611	0.152
Assist	Time (ref: pre-implementation)	1.153	0.225	<0.001
	ORIC subscale	4.395	1.911	0.022
	ORIC subscale*time	−2.190	2.454	0.373
Arrange	Time (ref: pre-implementation)	1.202	0.223	<0.001
	ORIC subscale	1.571	2.838	0.580
	ORIC subscale*time	−0.367	2.469	0.882

Note. Generalized linear mixed models were conducted to examine the moderation effect of organizational readiness for change on clinician screening and treatment behaviors between pre- and post-program implementation. * = multiplication operator to indicate the interaction term. ORIC = Organizational Readiness for Implementing Change. Ref = reference group in analyses. The number of total annual patient visits, number of total unique patient visits, and number of full-time employees were median-split and included as covariates in these analyses (not pictured above but available in Supplementary Table S3).

## Data Availability

The data presented in this study are available on request from the corresponding author. The data are not publicly available due to confidentiality/privacy agreements with funders.

## Supplementary Materials

The following are available online at https://www.mdpi.com/article/10.3390/ijerph181910485/s1, Table S1: Change in Clinician Screening and Treatment Behaviors Pre- to Post-Program Implementation by Substance Use Treatment Center (SUTC), Table S2: Adjusted Model of Organizational Demographics as Moderators of Clinician Screening and Treatment Behaviors Pre- to Post-Program Implementation, and Table S3: Adjusted Model of Organizational Readiness for Change Subscales as Moderators of Clinician Screening and Treatment Behaviors Pre- to Post-Program Implementation.
