# Supplementary material for: Organizational Factors Moderating Changes in Tobacco Use Dependence Care Delivery Following a Comprehensive Tobacco-Free Workplace Intervention in Non-Profit Substance Use Treatment Centers
Source: Int J Environ Res Public Health. 2021 Oct 6;18(19):10485. doi: 10.3390/ijerph181910485 (PMC8507614; doi:10.3390/ijerph181910485)
Supplement: Supplementary file 1 [file ijerph-18-10485-s001.zip › ijerph-1364757-supplementary.pdf]

Supplementary Table S1. Change in Clinician Screening and Treatment Behaviors Pre- to Post-Program Implementation by Substance Use Treatment Center (SUTC).

Clinician Behaviors	Pre-Implementation N	Pre Yes (%)	Post-Implementation N	Post Yes (%)	*p*-Value
**Ask**					
All SUTCs	*259*	*66.25*	*194*	*77.84*	*0.0036*
SUTC 1	11	45.45	3	100.00	0.2088
SUTC 2	65	43.08	50	70.00	0.0040
SUTC 3	6	100.00	4	100.00	NA
SUTC 4	23	91.30	19	89.47	1.0000
SUTC 5	30	83.33	14	100.00	0.1607
SUTC 6	25	76.00	22	81.82	0.7298
SUTC 7	11	63.64	13	92.31	0.1421
SUTC 8	9	88.89	7	100.00	1.0000
SUTC 9	3	100.00	2	100.00	NA
SUTC 10	3	66.67	4	100.00	0.4286
SUTC 11	3	66.67	2	100.00	1.0000
SUTC 12	8	100.00	1	100.00	NA
SUTC 13	50	52.00	40	52.50	0.9624
SUTC 14	3	100.00	5	80.00	1.0000
SUTC 15	9	66.67	8	87.50	0.5765
**Advise**					
All SUTCs	*223*	*60.54*	*181*	*71.82*	*0.0176*
SUTC 1	9	44.44	3	100.00	0.2045
SUTC 2	59	49.15	49	61.22	0.2096
SUTC 3	6	66.67	4	100.00	0.4667
SUTC 4	19	68.42	14	71.43	1.0000
SUTC 5	26	76.92	14	78.57	1.0000
SUTC 6	24	58.33	20	80.00	0.1244
SUTC 7	11	72.73	12	100.00	0.0932
SUTC 8	8	50.00	7	71.43	0.6084
SUTC 9	3	100.00	2	100.00	NA
SUTC 10	3	66.67	4	100.00	0.4286
SUTC 11	3	100.00	2	100.00	NA
SUTC 12	7	85.71	1	100.00	1.0000
SUTC 13	34	41.18	36	50.00	0.4589
SUTC 14	3	100.00	5	80.00	0.4076
SUTC 15	8	100.00	8	100.00	NA
**Assess**					
All SUTCs	*224*	*70.98*	*184*	*78.80*	*0.0712*
SUTC 1	9	55.56	3	100.00	0.4909
SUTC 2	59	49.15	48	72.92	0.0126
SUTC 3	6	83.33	4	100.00	1.0000
SUTC 4	20	95.00	17	94.12	1.0000
SUTC 5	26	96.15	13	92.31	1.0000
SUTC 6	24	83.33	21	76.19	0.7129
SUTC 7	11	63.64	13	84.62	0.3572
SUTC 8	8	87.50	7	100.00	1.0000
SUTC 9	3	100.00	2	100.00	NA
SUTC 10	3	100.00	4	100.00	NA
SUTC 11	3	33.00	2	100.00	0.4000
SUTC 12	7	100.00	1	100.00	NA

SUTC 13	34	50.00	36	61.11	0.3496
SUTC 14	3	100.00	5	60.00	0.4643
SUTC 15	8	100.00	8	87.50	1.0000
**Assist**					
All SUTCs	*222*	*45.95*	*181*	*69.61*	*<0.0001*
SUTC 1	9	33.33	3	100.00	0.1818
SUTC 2	60	30.00	49	65.31	0.0002
SUTC 3	6	83.33	4	100.00	1.0000
SUTC 4	19	42.11	14	57.14	0.3930
SUTC 5	26	84.62	14	85.71	1.0000
SUTC 6	23	56.52	20	85.00	0.0425
SUTC 7	11	36.36	13	69.23	0.1074
SUTC 8	7	57.14	7	71.43	1.0000
SUTC 9	3	100.00	2	100.00	NA
SUTC 10	3	0.00	4	75.00	0.1429
SUTC 11	3	66.67	2	100.00	1.0000
SUTC 12	7	57.14	1	100.00	1.0000
SUTC 13	34	29.41	35	51.43	0.0626
SUTC 14	3	100.00	5	60.00	0.4643
SUTC 15	8	37.50	8	87.50	0.1189
**Arrange**					
All SUTCs	*222*	*35.14*	*182*	*59.89*	*<0.0001*
SUTC 1	9	22.22	3	66.67	0.2364
SUTC 2	59	23.73	50	52.00	0.0023
SUTC 3	6	50.00	4	75.00	0.5714
SUTC 4	19	36.84	14	71.43	0.0799
SUTC 5	26	65.38	14	57.14	0.6070
SUTC 6	24	41.67	20	70.00	0.0755
SUTC 7	11	45.45	13	61.54	0.4307
SUTC 8	7	0.00	7	57.14	0.0699
SUTC 9	3	100.00	2	100.00	NA
SUTC 10	3	66.67	4	75.00	1.0000
SUTC 11	3	33.33	2	100.00	0.4000
SUTC 12	7	71.43	1	100.00	1.0000
SUTC 13	34	11.76	36	44.44	0.0025
SUTC 14	3	100.00	5	80.00	1.0000
SUTC 15	8	25.00	7	85.71	0.0406

Note. Chi-square tests/Fisher exact tests were conducted, as appropriate, to examine pre- to post-implementation changes in the 5 A's.

Supplementary Table S2. Adjusted Model of Organizational Demographics as Moderators of Clinician Screening and Treatment Behaviors Pre- to Post-Program Implementation.

**Clinician Behaviors**	**Number of total annual patient visits**			
	**Effect**	**Estimate**	**SE**	***p***
Ask	Time (ref: pre-implementation)	1.079	0.324	0.001
	Number of total annual patient visits	0.219	0.486	0.652
	Time*Number of total annual patient visits	-0.707	0.467	0.130
	ORIC overall	1.766	1.916	0.357
	Number of unique annual patient visits	0.663	0.733	0.366
	Number of full-time employees	-1.978	0.700	0.005
Advise	Time (ref: pre-implementation)	0.780	0.309	0.012
	Number of total annual patient visits	-0.211	0.465	0.651
	Time*Number of total annual patient visits	-0.329	0.457	0.471

	ORIC overall	3.437	1.650	0.038
	Number of unique annual patient visits	0.292	0.716	0.684
	Number of full-time employees	-0.763	0.611	0.212
Assess	Time (ref: pre-implementation)	0.823	0.335	0.015
	Number of total annual patient visits	0.462	0.602	0.443
	Time*Number of total annual patient visits	-0.601	0.506	0.236
	ORIC overall	-0.004	2.275	0.999
	Number of unique annual patient visits	0.336	0.919	0.715
	Number of full-time employees	-1.529	0.842	0.070
Assist	Time (ref: pre-implementation)	1.515	0.314	<0.001
	Number of total annual patient visits	-0.260	0.354	0.463
	Time*Number of total annual patient visits	-0.694	0.450	0.124
	ORIC overall	2.393	1.287	0.064
	Number of unique annual patient visits	-0.022	0.579	0.970
	Number of full-time employees	-1.098	0.502	0.029
Arrange	Time (ref: pre-implementation)	1.468	0.304	<0.001
	Number of total annual patient visits	0.116	0.321	0.719
	Time*Number of total annual patient visits	-0.555	0.438	0.206
	ORIC overall	3.974	1.272	0.002
	Number of unique annual patient visits	0.069	0.589	0.906
	Number of full-time employees	-0.605	0.507	0.233
	**Number of unique annual patient visits**			
	**Effect**	**Estimate**	**SE**	***p***
Ask	Time (ref: pre-implementation)	2.097	1.100	0.057
	Number of unique annual patient visits	0.967	0.761	0.204
	Time*Number of unique annual patient visits	-1.449	1.126	0.199
	ORIC overall	1.967	1.939	0.311
	Number of total annual patient visits	-0.040	0.455	0.929
	Number of full-time employees	-2.005	0.704	0.005
Advise	Time (ref: pre-implementation)	0.924	0.631	0.144
	Number of unique annual patient visits	0.413	0.738	0.576
	Time*Number of unique annual patient visits	-0.340	0.677	0.616
	ORIC overall	3.503	1.650	0.034
	Number of total annual patient visits	-0.347	0.427	0.417
	Number of full-time employees	-0.782	0.609	0.200
Assess	Time (ref: pre-implementation)	0.434	0.760	0.569
	Number of unique annual patient visits	0.329	0.947	0.728
	Time*Number of unique annual patient visits	0.148	0.804	0.854
	ORIC overall	0.074	2.294	0.974
	Number of total annual patient visits	0.227	0.573	0.692
	Number of full-time employees	-1.566	0.848	0.065
Assist	Time (ref: pre-implementation)	0.926	0.580	0.111
	Number of unique annual patient visits	-0.063	0.590	0.915
	Time*Number of unique annual patient visits	0.309	0.628	0.622
	ORIC overall	2.549	1.259	0.044
	Number of total annual patient visits	-0.553	0.294	0.061
	Number of full-time employees	-1.173	0.487	0.017
Arrange	Time (ref: pre-implementation)	0.479	0.480	0.319
	Number of unique annual patient visits	-0.219	0.603	0.717
	Time*Number of unique annual patient visits	0.914	0.541	0.092
	ORIC overall	4.036	1.256	0.001
	Number of total annual patient visits	-0.158	0.248	0.524

	Effect	Estimate	SE	*p*
	Number of full-time employees	-0.663	0.493	0.179

	Number of full-time employees			
	**Effect**	**Estimate**	**SE**	***p***
Ask	Time (ref: pre-implementation)	1.740	1.090	0.111
	Number of full-time employees	-1.794	0.716	0.013
	Time*Number of full-time employees	-1.058	1.116	0.344
	ORIC overall	2.015	1.929	0.297
	Number of total annual patient visits	-0.037	0.452	0.935
	Number of unique annual patient visits	0.714	0.727	0.326
Advise	Time (ref: pre-implementation)	0.672	0.570	0.239
	Number of full-time employees	-0.776	0.645	0.229
	Time*Number of full-time employees	-0.050	0.622	0.936
	ORIC overall	3.535	1.652	0.033
	Number of total annual patient visits	-0.345	0.426	0.418
	Number of unique annual patient visits	0.326	0.713	0.648
Assess	Time (ref: pre-implementation)	0.169	0.785	0.830
	Number of full-time employees	-1.715	0.904	0.058
	Time*Number of full-time employees	0.439	0.827	0.596
	ORIC overall	0.044	2.279	0.985
	Number of total annual patient visits	0.223	0.571	0.696
	Number of unique annual patient visits	0.365	0.923	0.693
Assist	Time (ref: pre-implementation)	0.585	0.533	0.273
	Number of full-time employees	-1.421	0.534	0.008
	Time*Number of full-time employees	0.725	0.586	0.217
	ORIC overall	2.393	1.259	0.058
	Number of unique annual patient visits	0.017	0.568	0.976
	Number of total annual patient visits	-0.566	0.295	0.056
Arrange	Time (ref: pre-implementation)	0.703	0.485	0.148
	Number of full-time employees	-0.946	0.552	0.087
	Time*Number of full-time employees	0.634	0.546	0.246
	ORIC overall	3.828	1.281	0.003
	Number of unique annual patient visits	0.122	0.588	0.835
	Number of total annual patient visits	-0.164	0.250	0.512

Note. Generalized linear mixed models were conducted to examine the moderation effect of organizational demographics on clinician screening and treatment behaviors from pre- and post-program implementation. ORIC=Organizational Readiness for Implementing Change. *=multiplication operator to indicate the interaction term. Ref=reference group in analyses. The number of total annual patient visits, number of total unique patient visits, and number of full-time employees were median-split.

Supplementary Table S3. Adjusted Model of Organizational Readiness for Change Subscales as Moderators of Clinician Screening and Treatment Behaviors Pre- to Post-Program Implementation.

Clinician		**ORIC Change Efficacy**		
Behaviors	**Effect**	**Estimate**	**SE**	***p***
Ask	Time (ref: pre-implementation)	0.815	0.239	0.001
	ORIC subscale	0.023	0.949	0.981
	ORIC subscale*time	1.638	0.728	0.025
	Number of total annual patient visits	0.156	0.459	0.734
	Number of unique annual patient visits	0.643	0.802	0.423
	Number of full-time employees	-2.156	0.756	0.005
Advise	Time (ref: pre-implementation)	0.639	0.230	0.006
	ORIC subscale	1.924	0.894	0.032
	ORIC subscale*time	0.272	0.702	0.698

	Number of total annual patient visits	0.119	0.398	0.766
	Number of unique annual patient visits	0.707	0.771	0.360
	Number of full-time employees	-1.255	0.657	0.057
Assess	Time (ref: pre-implementation)	0.569	0.252	0.024
	ORIC subscale	0.695	1.135	0.541
	ORIC subscale*time	0.440	0.784	0.574
	Number of total annual patient visits	0.298	0.534	0.577
	Number of unique annual patient visits	0.774	0.968	0.424
	Number of full-time employees	-1.816	0.875	0.039
Assist	Time (ref: pre-implementation)	1.190	0.227	<0.001
	ORIC subscale	0.102	0.711	0.886
	ORIC subscale*time	0.705	0.722	0.329
	Number of total annual patient visits	-0.379	0.317	0.233
	Number of unique annual patient visits	-0.365	0.641	0.570
	Number of full-time employees	-1.050	0.557	0.060
Arrange	Time (ref: pre-implementation)	1.260	0.229	<0.001
	ORIC subscale	2.287	0.843	0.007
	ORIC subscale*time	-1.292	0.808	0.110
	Number of total annual patient visits	0.268	0.303	0.377
	Number of unique annual patient visits	0.087	0.637	0.891
	Number of full-time employees	-0.870	0.567	0.126
**Clinician Behaviors**		**ORIC Change Commitment**		
	**Effect**	**Estimate**	**SE**	***p***
Ask	Time (ref: pre-implementation)	0.719	0.245	0.004
	ORIC subscale	2.918	0.650	<0.001
	ORIC subscale*time	-0.115	0.707	0.871
	Number of total annual patient visits	-1.069	0.328	0.001
	Number of unique annual patient visits	2.477	0.714	0.001
	Number of full-time employees	-3.080	0.656	<0.001
Advise	Time (ref: pre-implementation)	0.677	0.236	0.004
	ORIC subscale	2.124	0.603	<0.001
	ORIC subscale*time	0.564	0.656	0.391
	Number of total annual patient visits	-1.018	0.314	0.001
	Number of unique annual patient visits	0.905	0.623	0.147
	Number of full-time employees	-1.096	0.506	0.031
Assess	Time (ref: pre-implementation)	0.491	0.261	0.060
	ORIC subscale	1.720	1.203	0.154
	ORIC subscale*time	-0.581	0.717	0.418
	Number of total annual patient visits	-0.302	0.646	0.640
	Number of unique annual patient visits	1.412	1.067	0.187
	Number of full-time employees	-2.029	0.845	0.017
Assist	Time (ref: pre-implementation)	1.158	0.226	<0.001
	ORIC subscale	1.257	0.581	0.031
	ORIC subscale*time	-0.347	0.617	0.574
	Number of total annual patient visits	-0.813	0.305	0.008
	Number of unique annual patient visits	0.197	0.597	0.742
	Number of full-time employees	-1.324	0.503	0.009
Arrange	Time (ref: pre-implementation)	1.177	0.220	<0.001
	ORIC subscale	1.761	0.586	0.003
	ORIC subscale*time	-0.698	0.594	0.241
	Number of total annual patient visits	-0.525	0.288	0.069
	Number of unique annual patient visits	0.140	0.561	0.803

Clinician Behaviors	Effect	Estimate	SE	p
	Number of full-time employees	-0.717	0.459	0.119
**Clinician**		**ORIC Task Knowledge**		
**Behaviors**	**Effect**	**Estimate**	**SE**	***p***
Ask	Time (ref: pre-implementation)	0.802	0.244	0.001
	ORIC subscale	-1.021	0.349	0.004
	ORIC subscale*time	-0.658	0.605	0.278
	Number of total annual patient visits	0.108	0.227	0.635
	Number of unique annual patient visits	0.414	0.534	0.438
	Number of full-time employees	-2.261	0.614	<0.001
Advise	Time (ref: pre-implementation)	0.689	0.234	0.004
	ORIC subscale	-0.087	0.520	0.868
	ORIC subscale*time	-1.096	0.563	0.052
	Number of total annual patient visits	-0.024	0.378	0.949
	Number of unique annual patient visits	-0.662	0.590	0.263
	Number of full-time employees	-0.400	0.581	0.492
Assess	Time (ref: pre-implementation)	0.509	0.258	0.049
	ORIC subscale	-1.515	0.476	0.002
	ORIC subscale*time	0.350	0.616	0.570
	Number of total annual patient visits	0.222	0.373	0.553
	Number of unique annual patient visits	0.273	0.652	0.676
	Number of full-time employees	-1.960	0.729	0.008
Assist	Time (ref: pre-implementation)	1.178	0.225	<0.001
	ORIC subscale	-0.231	0.325	0.477
	ORIC subscale*time	-0.474	0.509	0.353
	Number of total annual patient visits	-0.377	0.223	0.092
	Number of unique annual patient visits	-0.642	0.474	0.176
	Number of full-time employees	-0.962	0.476	0.044
Arrange	Time (ref: pre-implementation)	1.187	0.223	<0.001
	ORIC subscale	-0.319	0.422	0.450
	ORIC subscale*time	-0.112	0.499	0.822
	Number of total annual patient visits	0.071	0.302	0.815
	Number of unique annual patient visits	-0.944	0.473	0.047
	Number of full-time employees	-0.282	0.466	0.545
**Clinician**		**ORIC Resource Availability**		
**Behaviors**	**Effect**	**Estimate**	**SE**	***p***
Ask	Time (ref: pre-implementation)	0.791	0.239	0.001
	ORIC subscale	0.645	0.538	0.232
	ORIC subscale*time	-1.666	0.710	0.019
	Number of total annual patient visits	-0.018	0.470	0.970
	Number of unique annual patient visits	0.051	0.830	0.951
	Number of full-time employees	-1.694	0.836	0.043
Advise	Time (ref: pre-implementation)	0.656	0.231	0.005
	ORIC subscale	0.400	0.518	0.441
	ORIC subscale*time	-1.033	0.648	0.112
	Number of total annual patient visits	-0.116	0.460	0.801
	Number of unique annual patient visits	-0.745	0.718	0.299
	Number of full-time employees	-0.271	0.699	0.698
Assess	Time (ref: pre-implementation)	0.585	0.259	0.024
	ORIC subscale	0.758	0.615	0.218
	ORIC subscale*time	-1.923	0.747	0.010
	Number of total annual patient visits	0.142	0.555	0.799
	Number of unique annual patient visits	-0.054	0.929	0.954

	Number of full-time employees	-1.208	0.926	0.193
Assist	Time (ref: pre-implementation)	1.188	0.226	<0.001
	ORIC subscale	0.899	0.391	0.022
	ORIC subscale*time	-0.963	0.573	0.093
	Number of total annual patient visits	-0.478	0.265	0.072
	Number of unique annual patient visits	-1.059	0.531	0.047
	Number of full-time employees	-0.586	0.497	0.239
Arrange	Time (ref: pre-implementation)	1.208	0.223	<0.001
	ORIC subscale	0.652	0.413	0.115
	ORIC subscale*time	-0.366	0.544	0.502
	Number of total annual patient visits	0.056	0.313	0.859
	Number of unique annual patient visits	-1.224	0.557	0.029
	Number of full-time employees	-0.011	0.539	0.984
**Clinician Behaviors**		**ORIC Change Valence**		
	**Effect**	**Estimate**	**SE**	***p***
Ask	Time (ref: pre-implementation)	0.698	0.236	0.003
	ORIC subscale	6.488	3.282	0.049
	ORIC subscale*time	-2.930	2.452	0.233
	Number of total annual patient visits	-0.250	0.461	0.588
	Number of unique annual patient visits	0.673	0.690	0.330
	Number of full-time employees	-2.025	0.735	0.006
Advise	Time (ref: pre-implementation)	0.648	0.232	0.005
	ORIC subscale	3.686	3.783	0.331
	ORIC subscale*time	1.029	2.416	0.670
	Number of total annual patient visits	-0.258	0.464	0.578
	Number of unique annual patient visits	-0.512	0.647	0.430
	Number of full-time employees	-0.324	0.644	0.616
Assess	Time (ref: pre-implementation)	0.474	0.256	0.065
	ORIC subscale	6.724	4.181	0.109
	ORIC subscale*time	-3.745	2.611	0.152
	Number of total annual patient visits	-0.046	0.552	0.934
	Number of unique annual patient visits	0.554	0.798	0.488
	Number of full-time employees	-1.528	0.816	0.062
Assist	Time (ref: pre-implementation)	1.153	0.225	<0.001
	ORIC subscale	4.395	1.911	0.022
	ORIC subscale*time	-2.190	2.454	0.373
	Number of total annual patient visits	-0.706	0.274	0.010
	Number of unique annual patient visits	-0.503	0.488	0.303
	Number of full-time employees	-0.897	0.488	0.067
Arrange	Time (ref: pre-implementation)	1.202	0.223	<0.001
	ORIC subscale	1.571	2.838	0.580
	ORIC subscale*time	-0.367	2.469	0.882
	Number of total annual patient visits	0.040	0.358	0.911
	Number of unique annual patient visits	-0.880	0.520	0.092
	Number of full-time employees	-0.248	0.515	0.630

Note. Generalized linear mixed models were conducted to examine the moderation effect of organizational readiness for change on clinician screening and treatment behaviors from pre- and post-program implementation. ORIC=Organizational Readiness for Implementing Change. *=multiplication operator to indicate the interaction term Ref=reference group in analyses. The number of total annual patient visits, number of total unique patient visits, and number of full-time employees were median-split.
